# Comparison of Five Serological Assays for the Detection of SARS-CoV-2 Antibodies

**DOI:** 10.3390/diagnostics11010078

**Published:** 2021-01-06

**Authors:** Anja Dörschug, Julian Schwanbeck, Andreas Hahn, Anke Hillebrecht, Sabine Blaschke, Kemal Mese, Uwe Groß, Sascha Dierks, Hagen Frickmann, Andreas E. Zautner

**Affiliations:** 1 Institute for Medical Microbiology, University Medical Center Göttingen, 37075 Göttingen, Germany; anja.doerschug@stud.uni-goettingen.de (A.D.); julian.schwanbeck@med.uni-goettingen.de (J.S.); kemal.mese@med.uni-goettingen.de (K.M.); ugross@gwdg.de (U.G.); 2 Institute for Medical Microbiology, Virology and Hygiene, University Medicine Rostock, 18057 Rostock, Germany; andreas.hahn@uni-rostock.de (A.H.); hagen.frickmann@med.uni-rostock.de (H.F.); 3 Interdisciplinary Emergency Department, University Medical Center Göttingen, 37075 Göttingen, Germany; anke.hillebrecht@med.uni-goettingen.de (A.H.); sblasch@gwdg.de (S.B.); 4 Institute for Clinical Chemistry, University Medical Center Göttingen, 37075 Göttingen, Germany; sascha.dierks@med.uni-goettingen.de; 5 Department of Microbiology and Hospital Hygiene, Bundeswehr Hospital Hamburg, 20359 Hamburg, Germany

**Keywords:** SARS-CoV-2, COVID-19, serology, test comparison, surveillance

## Abstract

Serological assays can contribute to the estimation of population proportions with previous immunologically relevant contact with the Severe Acute Respiratory Syndrome Corona Virus 2 (SARS-CoV-2) virus. In this study, we compared five commercially available diagnostic assays for the diagnostic identification of SARS-CoV-2-specific antibodies. Depending on the assessed immunoglobulin subclass, recorded sensitivity ranged from 17.0% to 81.9% with best results for immunoglobulin G. Specificity with blood donor sera ranged from 90.2% to 100%, with sera from EBV patients it ranged from 84.3% to 100%. Agreement from fair to nearly perfect was recorded depending on the immunoglobulin class between the assays, the with best results being found for immunoglobulin G. Only for this immunoglobulin class was the association between later sample acquisition times (about three weeks after first positive PCR results) and positive serological results in COVID-19 patients confirmed. In conclusion, acceptable and comparable reliability for the assessed immunoglobulin G-specific assays could be shown, while there is still room for improvement regarding the reliability of the assays targeting the other immunoglobulin classes.

## 1. Introduction

The Corona Virus Disease 2019 (COVID-19) pandemic, caused by Severe Acute Respiratory Syndrome Corona Virus 2 (SARS-CoV-2) and starting in Wuhan, China, in 2019 [1], remains the most threatening global public health menace of the year 2020. On a global scale, diagnosis, containment and surveillance of the disease were considered issues of major concern.

For containment purposes, direct proof of virus RNA in respiratory samples is of central importance, so molecular tools for the detection of SARS-CoV-2 virus RNA were rapidly introduced and evaluated [2,3,4,5,6,7,8,9,10,11,12,13,14] at early stages of the pandemic. However, detectable amounts of virus RNA can quickly decline over the course of the disease [12,15], so infected individuals with lacking or mild symptoms have a good chance of going undetected if surveillance is just based on molecular diagnostic approaches.

To close this diagnostic gap, there was an early focus on the implementation of antibody-based surveillance. By doing so, a more realistic view on the real dimensions of the spread of SARS-CoV-2 in the population was aspired to and numerous benchtop-based and point-of-care-testing (POCT)-based serological assays were introduced [16,17,18,19,20,21,22,23,24,25,26,27,28,29,30,31,32,33,34,35,36,37,38,39,40,41,42,43,44,45,46,47]. However, the limitations of this strategy rapidly emerged as well. While specificity was usually at least >95% in the geographic regions where the tests were developed, a broad variety of sensitivities, usually between 70% and 90% depending on the subpopulation assessed [16,17,18,19,20,21,22,23,24,25,26,27,28,29] and sometimes even lower [47], were recorded with an optimum sensitivity two weeks after infection [29] and decreasing positivity rates afterwards [30]. Further, age-dependency of serological sensitivity has been demonstrated [33] next to higher specificity but lower sensitivity of neutralizing antibodies compared to non-neutralizing ones [34].

More than this, it has become obvious that immunologically relevant contacts with SARS-CoV-2, i.e., viral in-vivo replication leading to any adaptive immune response, can occur completely without the induction of specific antibodies but just with SARS-CoV-2-specific T cell responses [48]. This is well in line with observed low seropositivity in patients with previous PCR-confirmed COVID-19 as observed in a recent study [47]. It is discussed that specific T cells may provide protection against SARS-CoV-2 even in the absence of antibodies [49].

However, the method-immanent imperfect sensitivity of diagnostic methods does not necessarily mean that they cannot be used for surveillance purposes. If diagnostic accuracy adjusted methods [50,51] are applied, the true prevalence can be estimated even based on a test with imperfect but known test characteristics in epidemiological assessments. Accordingly, the evaluation of test characteristics of serological tests for antibodies against SARS-CoV-2 is still an issue of epidemiological relevance.

In the study performed here, five commercially available serological assays targeting SARS-CoV-2-specific antibodies were assessed. The comparison comprised previously described products such as the assays from EUROIMMUN (Lübeck, Germany) [26,27,35,36,37,38,39,40,41,42,43,44,45,46,47,52,53], Roche (Basel, Switzerland) [54,55,56,57,58,59], Mikrogen (Neuried, Germany) [60,61,62], and Virotech Diagnostics (Rüsselsheim am Main, Germany) [46,55,63] as well as a newly evaluated kit from Vircell (Vircell, Granada, Spain). As positive controls, residual serum samples from patients with PCR-confirmed COVID-19 were used, while samples from blood donors and patients with Epstein–Barr virus (EBV) were applied as negative controls. With this approach, test characteristics should be calculated to guide the application of the serological assays for both diagnostic and surveillance purposes. Surveillance purposes include population prevalence studies and diagnostic purposes include the confirmation of previous infections with SARS-CoV-2 in individual patients.

## 2. Materials and Methods

### 2.1. Sample Collections

In the same way as described before [47], three different serum sample collections were assessed comprising one collection of positive controls and two negative control collections. The positive control sample collection consisted of samples from 148 PCR-confirmed COVID-19 patients, on whom PCR had been performed from nasopharyngeal swabs. Due to limited sample volumes, between 100 and 148 samples were assessed with each assessed serological assay. For those samples from PCR-confirmed COVID-19 patients, the time between the positive PCR result and the acquisition of the serum samples was documented in 94 out of 148 (63.5%) instances, with time periods ranging from −2 to 120 days (median: 11 days, mean: 20.3 days, standard deviation (SD): 24.2 days). The 54 specimen donors with no clearly documented time between the positive PCR result and the acquisition of the serum sample for data protection reasons were candidates for convalescent serum donation who were referred via the Department of Transfusion Medicine of the University Medical Center Göttingen. However, the time between the positive PCR result and the acquisition of the serum sample from these specimens is at least 4 weeks.

The first negative control collection consisted of samples from 152 blood donors acquired in 2015 and thus well before the COVID-19 pandemic began. Sufficient sample volumes were available to allow testing of 50 to 152 out of those negative controls per assay. The second negative control collection contained 32 Epstein–Barr virus (EBV)-positive serum samples, which had been collected at the beginning of 2020 when the likelihood of COVID-19 infections was still extremely low in Germany. Sufficient volumes for the testing of 30 to 32 samples out of those second negative control population per test assay were available. This third serum collection was included to assess the effects of polyclonal B cell stimulation. The used sample volumes were residual sample materials from routine diagnostic procedures performed at the University Medical Center Göttingen.

As the ethical board allowed only a completely anonymized use of sample materials for test comparison purposes, no patient-specific information can be provided, necessarily resulting in an unavoidable violation of the Standards for Reporting of Diagnostic Accuracy (STARD) criteria [64].

### 2.2. Serological Assays

The compared serological assays comprised:

- The EUROIMMUN COVID-19 IgG/IgA assay (EUROIMMUN, Lübeck, Germany; referred to as “EUROIMMUN assay” in the following);
- The Roche Cobas Elecsys Anti-SARS-CoV-2 assay (Roche, Basel, Switzerland; referred to as “Roche assay” in the following);
- The Mikrogen *recom*Well SARS-CoV-2 IgG assay (Neuried, Germany; referred at as “Mikrogen assay” in the following);
- The Virotech Diagnostics assay VIROTECH SARS-CoV-2 IgA/IgM/IgG ELISA (Rüsselsheim am Main, Germany; referred to as “Virotech assay” in the following);
- The Vircell COVID-19 ELISA IgG/IgM+IgA assay (Vircell, Grenada, Spain; referred to as Vircell assay in the following).

All assays were exactly performed as demanded by the manufacturers’ instructions.

### 2.3. Real-Time PCR Testing

To characterize the positive control samples, respiratory sample materials from the patients with suspected or confirmed COVID-19 were analyzed using real-time PCR for SARS-CoV-2 in a two-step procedure. In step one, screening was performed applying the Genesig Real-Time PCR Coronavirus (COVID-19) assay (Primerdesign Ldt., Chandlers Ford, UK). In a second step, first-time positive results were confirmed using automated Cepheid Xpert Xpress SARS-CoV-2 PCR (Cepheid, Sunnyvale, CA, USA). Both SARS-CoV-2-specific PCR assays were performed exactly as described by the manufacturers.

### 2.4. Statistical Assessment

Due to the restricted number of samples, descriptive statistical analysis was performed only. With the positive control sample collection taken from patients with PCR-confirmed COVID-19, sensitivity was calculated. To assess the effect of the number of days between positive PCR results and the serum sample acquisition, Wilcoxon rank sum testing was calculated applying the software Stata/IC 15.1 for macOS 64-bit Intel (College Station, TX, USA).

With the two negative control sample collections taken from the blood donors and the EBV patients, specificity of the serological assays was assessed. Positive and negative predictive values were calculated for two exemplary populations with 1% and 10% prevalence, respectively.

Cohen’s kappa [65] was calculated to assess the agreement between the test assays with the categories of poor (below 0.00), slight (0.00–0.20), fair (0.21–0.40), moderate (0.41–0.60), substantial (0.61–0.80) and almost perfect (0.81–1.00) for the immunoglobulin subclasses as well as across classes targeting the meta-structure “any SARS-CoV-2-related antibodies”.

In line with common conventions of descriptive statistics, standard deviation (SD) was calculated for mean values and interquartile range (IQR) for median values. Additional calculation of median values next to mean values was performed to indicate left- or right-shifted distributions of values within the different groups—i.e., information which would have gone unreported otherwise.

Samples were not excluded if individual data points were missing due to insufficient amounts of sample material as stated above.

### 2.5. Ethics

The study was ethically approved by the institutional ethics board of the University Medical Center Göttingen (Application number 21/05/20), allowing the use of residual sample materials for test comparison purposes.

## 3. Results

### 3.1. Calculated Sensitivity

Assessed by immunoglobulin classes, observed sensitivities of the evaluated test assays as recorded exclusively with samples from patients with previous positive results of SARS-CoV-2 PCR differed considerably. For IgG, sensitivities ranged from 63.0% to 81.9%, for IgA from 21.0% to 81.8%, for IgM from 17.0% to 20.0% and for the overarching assays measuring different immunoglobulin classes from 66.6% to 76.1%. The values slightly varied depending on whether borderline results were interpreted as positive or as negative. Details of individual assays are provided in Table 1.

### 3.2. Influence of the Time between Positive PCR Results and Serum Sample Acquisition

Significance for higher likelihood of detecting positive signals after prolonged duration of about 3 weeks between the first recorded positive PCR result and serum acquisition was calculated for all IgG-specific assays and Roche’s immunoglobulin class-overarching assay. When focusing on the median instead of the mean numbers of days, significance for higher likelihood of positive results for SARS-CoV-2-specific IgG was detectable after about two weeks in most instances, indicating a left-shifted distribution of the recorded values. In contrast, the Virotech IgA assay was associated with a higher reliability after a short time period of little more than a week. For the other assays targeting IgA or IgM, no time-dependency could be confirmed. Details are provided in Table 2.

### 3.3. Calculated Specificity Based on Blood Donor Samples as Negative Contol Samples

Recorded specificity with blood donor samples, which had been collected prior to the COVID-19 pandemic, used as negative control samples ranged from 90.2% to 100%. Recorded specificities <95% were seen for the Mikrogen IgG assay only if borderline results were counted as positives. For the EUROIMMUN IgA assay, the Vircell IgM/IgA assay and Virotech IgM assay, specificity remained below this threshold even if borderline samples were considered as negative. Details are provided in Table 3.

### 3.4. Calculated Specificity Based on Samples from EBV-Positive Patients

Recorded specificity with samples from EBV-positive patients collected at the very beginning of the COVID-19 pandemic (used as negative samples) ranged from 84.3% to 100%. Recorded specificities <95% were seen for the Mikrogen IgG assay and the Vircell IgG assay only if borderline results were counted as positives. For the Vircell IgM/IgA assay, specificity remained below 90% even if borderline samples were considered negative. Details are provided in Table 4.

### 3.5. Positive and Negative Predictive Values as Calculated for Exemplary Populations with 1% and 10% Prevalence

Based on the results as shown above, positive and negative predictive values were calculated in a mathematical modelling for two hypothetical exemplary populations with 1% and 10% prevalence of SARS CoV 2-specific antibodies. Over the different assessed assays, the negative predictive value was excellent with 99.1% till 99.8% for the 1% prevalence population but dropped to 91.3% till 97.9% for the 10% prevalence population. In contrast, for the 1% prevalence population, positive predictive values ranged from 3.9% till 100%, while this range was narrowed to 30.7% till 100% for the 10% prevalence population. Details are provided in Table 5.

### 3.6. Agreement Kappa

Almost perfect agreement (0.81–1.00) between the compared assays according to the definitions by Landis and Koch [65] was observed for the IgG immunoglobulin class only. For immunoglobulin class-overarching comparisons, only moderate (0.41–0.60) to substantial (0.61–0.80) agreement could be seen with worse results if IgM was included. For the IgA immunoglobulin class, agreement even dropped to the fair level (0.21–0.40). Details are provided in Table 6.

## 4. Discussion

The study was conducted to provide information on performance characteristics of commercially available serological assays. Thus, it contributes to previously described assessments [16,17,18,19,20,21,22,23,24,25,26,27,28,29,30,31,32,33,34,35,36,37,38,39,40,41,42,43,44,45,46,47,52,53,54,55,56,57,58,59,60,61,62,63] and provides an additional piece of the puzzle in terms of interpreting the results of serological approaches for the retrospective diagnosis of infections with SARS-CoV-2.

One major result of the study is the confirmation of acceptable sensitivity and good specificity, associated with nearly perfect agreement, for the assays detecting SARS-CoV-2 antibodies of the immunoglobulin class IgG. While interpreting the less than perfect sensitivity, one has to consider the variance in the periods of time between the first recorded positive SARS-CoV-2 PCRs and sample acquisition for serological assessments. In case of longer periods of about 3 weeks, all IgG assays scored significantly better than in the case of shorter periods. This is well in line with previous reports [29]. Further, detectable immunoglobulins are not always detectable in individuals with confirmed immunologically relevant contact with SARS-CoV-2 [47,48,49]. Though a more detailed assessment of clinical data of SARS-CoV-2 infected patients without recorded seroconversion would have been desirable, the strict focus of the study design on the test comparison prevented this option, an undeniable limitation of this approach.

Focusing on other immunoglobulin classes such as IgM and IgA, the performance characteristics of the assessed assays were considerably worse, also confirming previous results [47]. This phenomenon was shown to affect sensitivity, specificity and also intertest agreement. A comparably good specificity as observed for the Virotech IgM and IgA assays was traded for particular poor sensitivity in these assays, while the other test producers seem to have aimed at a compromise between sensitivity and specificity. Thereby, sensitivity of the Virotech IgA assay was better in the early stages of infection about one week after the first positive PCR test, a phenomenon which could be shown for no other assay.

Polyclonal B-cell proliferation, as associated with EBV infection [66], particularly affected the Vircell IgM/IgG assay. For the other assays, specificity with sera from blood donors and with sera from EBV patients was quite comparable.

The study has a number of limitations beyond the one stated above. Firstly, limited volumes of residual sample materials did not allow the assessment of all samples with all assays. Secondly, ethical considerations did not allow the inclusion of patient data which is an undeniable violation of the recommendations by the STARD guideline [64]. Thirdly, the assessed assays are not representative of all respective products available on the market. Fourthly, not all immunoglobulin classes were represented with equal numbers of assays, preventing the calculation of Cohen’s kappa for immunoglobulin class M. Fifthly, economic restrictions limited the assessments to reasonable but still low sample numbers.

In spite of these limitations, the study provides another piece in the diagnostic puzzle, allowing a better interpretation of results of serological assays targeting antibodies against SARS-CoV-2.

## 5. Conclusions

This study indicates acceptable reliability of immunoglobulin class G-based serology for SARS-CoV-2-specific antibodies with a variety of test assays with increased sensitivities about 3 weeks after first positive PCR results compared with earlier time points. Assays for other immunoglobulin classes scored worse with less obvious associations to the time points of testing.

## Figures and Tables

**Table 1:** Sensitivities of the assessed assays.

Test	*N*	Positives ^1^	Sensitivity (0.95 CI)	Positives ^2^	Sensitivity (0.95 CI)
EUROIMMUN assay IgA	148	121	0.818 (0.746, 0.872)	110	0.743 (0.666, 0.808)
EUROIMMUN assay IgG	148	120	0.811 (0.739, 0.866)	120	0.811 (0.739, 0.866)
Mikrogen assay IgG	105	82	0.780 (0.690, 0.850)	79	0.752 (0.659, 0.826)
Vircell assay IgG	105	86	0.819 (0.732, 0.882)	84	0.800 (0.711, 0.866)
Vircell assay IgM/IgA	105	79	0.752 (0.659, 0.826)	70	0.666 (0.570, 0.750)
Roche assay	105	80	0.761 (0.670, 0.834)	80	0.761 (0.670, 0.834)
Virotech assay IgA	100	23	0.230 (0.157, 0.324)	21	0.210 (0.140, 0.302)
Virotech assay IgG	100	71	0.710 (0.613, 0.791)	63	0.630 (0.530, 0.720)
Virotech assay IgM	100	20	0.200 (0.132, 0.291)	17	0.170 (0.108, 0.258)

^1^ Borderline results were counted as positive. ^2^ Borderline results were counted as negative. *N* = numbers. CI = confidence interval. IgA/G/M = immunoglobulin A/G/M.

**Table 2:** Comparison of the time (in days) between positive PCR results and serum sample acquisition of the different assays.

Test	*N*	Positives	Days Mean (SD) Median (IQR)	Negatives	Days Mean (SD) Median (IQR)	*p* Value *
EUROIMMUN assay IgA	88	74	19.27 (21.44) 12.5 (4, 29)	14	23.21 (37.43) 3.5 (2, 29)	0.2755
EUROIMMUN assay IgG	94	74	24.08 (25.38) 16.5 (6, 35)	20	6.10 (10.96) 3 (1.5, 6)	0.0001
Mikrogen assay IgG	68	54	21.31 (18.63) 16 (7, 35)	14	9.36 (14.56) 3.5 (2, 9)	0.0086
Vircell assay IgG	70	57	20.37 (18.59) 15 (4, 34)	13	7.31 (13.39) 3 (2, 4)	0.0059
Vircell assay IgM/IgA	66	51	16.49 (15.80) 13 (3, 22)	15	17.20 (21.22) 4 (2, 36)	0.5865
Roche assay	71	54	21.33 (18.61) 16 (7, 35)	17	8.06 (13.46) 3 (1, 8)	0.0014
Virotech assay IgA	58	14	8.07 (12.44) 4.5 (1, 7)	44	30.02 (30.51) 21.5 (3.5, 48)	0.0098
Virotech assay IgG	55	36	27.97 (26.54) 21.5 (6, 38.5)	19	12.42 (20.15) 4 (1, 11)	0.0047
Virotech assay IgM	58	12	9.08 (12.83) 3.5 (3, 9)	46	28.37 (30.44) 20.5 (4, 48)	0.0571

* Wilcoxon ranksum test; *N* = number; mean = arithmetic mean (average); SD = standard deviation; median = middle value separating the greater and lesser halves of a data set; IQR = interquartile range; IgA/G/M = immunoglobulin A/G/M.

**Table 3:** Specificity of the test assays as calculated based on the blood donor sera.

Test	*N*	Negatives ^1^	Specificity (0.95 CI)	Negatives ^2^	Specificity (0.95 CI)
EUROIMMUN assay IgA	152	142	0.934 (0.882, 0.964)	144	0.947 (0.898, 0.974)
EUROIMMUN assay IgG	152	150	0.989 (0.948, 0.997)	152	1 (n.e.)
Mikrogen assay IgG	102	95	0.931 (0.862, 0.967)	102	1 (n.e.)
Vircell assay IgG	102	100	0.980 (0.924, 0.995)	101	0.990 (0.932, 0.999)
Vircell assay IgM/IgA	102	92	0.902 (0.826, 0.947)	94	0.922 (0.850, 0.961)
Roche assay	102	102	1 (n.e.)	102	1 (n.e.)
Virotech assay IgA	100	100	1 (n.e.)	100	1 (n.e.)
Virotech assay IgG	100	100	1 (n.e.)	100	1 (n.e.)
Virotech assay IgM	50	47	0.940 (0.826, 0.981)	47	0.940 (0.826, 0.981)

^1^ Borderline results were counted as positive; ^2^ Borderline results were counted as negative; *N* = numbers; CI = confidence interval; IgA/G/M = immunoglobulin A/G/M; n.e. = not estimable.

**Table 4:** Specificity of the test assays as calculated based on the sera from the Epstein–Barr virus (EBV) patients.

Test	*N*	Negatives ^1^	Specificity (0.95 CI)	Negatives ^2^	Specificity (0.95 CI)
EUROIMMUN assay IgA	32	31	0.968 (0.796, 0.995)	31	0.968 (0.796, 0.995)
EUROIMMUN assay IgG	32	31	0.968 (0.796, 0.995)	31	0.968 (0.796, 0.995)
Mikrogen assay IgG	32	30	0.937 (0.771, 0.985)	31	0.968 (0.796, 0.995)
Vircell assay IgG	32	30	0.937 (0.771, 0.985)	31	0.968 (0.796, 0.995)
Vircell assay IgM/IgA	32	27	0.843 (0.666, 0.935)	28	0.875 (0.701, 0.954)
Roche assay	32	32	1 (n.e.)	32	1 (n.e.)
Virotech assay IgA	30	30	1 (n.e.)	30	1 (n.e.)
Virotech assay IgG	30	30	1 (n.e.)	30	1 (n.e.)
Virotech assay IgM	30	29	0.967 (0.784, 0.996)	29	0.967 (0.784, 0.996)

^1^ Borderline results were counted as positive; ^2^ borderline results were counted as negative; *N* = numbers; CI = confidence interval; IgA/G/M = immunoglobulin A/G/M; n.e. = not estimable.

**Table 5:** Positive and negative predictive values * as calculated for two exemplary populations with 1% and 10% prevalence, respectively.

Test	Prevalence 1%		Prevalence 10%	
	PPV	NPV	PPV	NPV
EUROIMMUN assay IgA	0.147	0.998	0.655	0.975
EUROIMMUN assay IgG	0.304	0.998	0.828	0.979
Mikrogen assay IgG	0.159	0.998	0.675	0.974
Vircell assay IgG	0.207	0.998	0.742	0.979
Vircell assay IgM/IgA	0.059	0.997	0.408	0.965
Roche assay	1	0.998	1	0.974
Virotech assay IgA	1	0.992	1	0.920
Virotech assay IgG	1	0.997	1	0.965
Virotech assay IgM	0.039	0.991	0.307	0.913

* All sensitivities and specificities are weighted equally; PPV = positive predictive value; NPV = negative predictive value.

**Table 6:** Agreement between the tests by immunoglobulin classes and in overarching assessments.

Test Groups	*N*	Kappa	0.95 CI
IgA ^1^	160	0.220	(0.142, 0.329)
IgG ^2^	67	0.803	(0.734, 0.886)
IgA/IgG/IgM/Roche ^3^	57	0.509	(0.445, 0.593)
IgA/IgG/Roche ^3^	142	0.721	(0.748, 0.800)

^1^ EUROIMMUN IgA assay, Virotech IgA assay; ^2^ EUROIMMUN IgG assay, Mikrogen IgG assay, Virotech IgG assay, Vircell IgG assay; ^3^ immunoglobulin class positive, if at least one assay for this class shows a positive result; immunoglobulin class negative, if at least one assay for this class shows negative result and no other test shows positive result; immunoglobulin class uncertain, if at least one test with respective specificity is borderline and others are neither positive nor negative; *N* = numbers; CI = confidence interval; IgA/G/M = immunoglobulin A/G/M.

## Data Availability

All relevant data are provided in the manuscripts and its tables.
